# Metagenomic analysis of hot spring soil for mining a novel thermostable enzybiotic

**DOI:** 10.1007/s00253-023-12979-2

**Published:** 2024-01-22

**Authors:** Panagiota D. Pantiora, Nikolaos D. Georgakis, Georgios E. Premetis, Nikolaos E. Labrou

**Affiliations:** Laboratory of Enzyme Technology, Department of Biotechnology, School of Applied Biology and Biotechnology, Agricultural University of Athens, 75 Iera Odos Street, GR-11855 Athens, Greece

**Keywords:** Enzybiotics, Endolysins, Lytic enzymes, Antibiotic-resistant pathogens, Antimicrobials, Antibiotics

## Abstract

**Abstract:**

The misuse and overuse of antibiotics have contributed to a rapid emergence of antibiotic-resistant bacterial pathogens. This global health threat underlines the urgent need for innovative and novel antimicrobials. Endolysins derived from bacteriophages or prophages constitute promising new antimicrobials (so-called enzybiotics), exhibiting the ability to break down bacterial peptidoglycan (PG). In the present work, metagenomic analysis of soil samples, collected from thermal springs, allowed the identification of a prophage-derived endolysin that belongs to the *N*-acetylmuramoyl-L-alanine amidase type 2 (NALAA-2) family and possesses a LysM (lysin motif) region as a cell wall binding domain (CWBD). The enzyme (Ami1) was cloned and expressed in *Escherichia coli*, and its bactericidal and lytic activity was characterized. The results indicate that Ami1 exhibits strong bactericidal and antimicrobial activity against a broad range of bacterial pathogens, as well as against isolated peptidoglycan (PG). Among the examined bacterial pathogens, Ami1 showed highest bactericidal activity against *Staphylococcus aureus s*and *Staphylococcus epidermidis* cells. Thermostability analysis revealed a melting temperature of 64.2 ± 0.6 °C. Overall, these findings support the potential that Ami1, as a broad spectrum antimicrobial agent, could be further assessed as enzybiotic for the effective treatment of bacterial infections.

**Key points:**

• *Metagenomic analysis allowed the identification of a novel prophage endolysin*

• *The endolysin belongs to type 2 amidase family with lysin motif region*

• *The endolysin displays high thermostability and broad bactericidal spectrum*

**Supplementary information:**

The online version contains supplementary material available at 10.1007/s00253-023-12979-2.

## Introduction

Antimicrobial pharmaceutical products have undoubtedly saved millions of lives worldwide. However, their uncontrolled prescription and consumption in combination with their misuse have contributed to the increasing emergence of multidrug-resistant (MDR) bacterial pathogens (Danis-Wlodarczyk et al. 2021). This global health crisis is emphasized as an unprecedented threat to human health by the World Health Organization (WHO) and the European Society of Clinical Microbiology and Infectious Diseases (ESCMID) (Harhala et al. 2021; Laxminarayan et al. 2013; Murray et al. 2021).

The rapid resistance against a multitude of antibiotic substances acquired by the bacterial species *Enterococcus faecium*, *Staphylococcus aureus*, *Klebsiella pneumoniae*, *Acinetobacter baumannii*, *Pseudomonas aeruginosa*, and the *Enterobacteriaceae* species (ESKAPE pathogens) renders them leading factors of morbidity and mortality, thus threatening both public and animal health (Laxminarayan et al. 2013). This phenomenon combined with the limited development of new and effective antibiotics and treatments by the pharmaceutical industry has led research into the discovery and production of alternative antibacterial agents that act with innovative mechanisms, thus minimizing or even eliminating resistance development (Danis-Wlodarczyk et al. 2021).

Towards this direction, the utilization of enzybiotics, a specific class of enzymes that exhibit the potency to treat bacterial infections (Labrou 2019; Song et al. 2020), is a very promising and innovative approach (Harhala et al. 2021; Varotsou et al. 2023). The antimicrobial potential of enzybiotics lies heavily on their ability to hydrolyze PG, a fundamental structural element of the bacterial cell wall, causing cell lysis and therefore its death (Young 1992; Fischetti 2001). Enzybiotics have received much attention as potential antibacterial agents, as they present a wide range of desirable characteristics and properties. Main features of enzybiotics include (i) a rapid and unique mechanism of action against pathogenic bacteria, acting independently of the host’s active metabolism (Schmelcher et al. 2012); (ii) high specificity for killing pathogens (Schmelcher et al. 2012; Rodríguez-Rubio et al. 2013); (iii) low probability of developing bacterial resistance (Schmelcher et al. 2012); (iv) selectivity against gram-negative and gram-positive pathogens, which can be further enhanced through protein engineering; (v) activity against mycobacteria; (vi) bactericidal action (Hermoso et al. 2003; Kashani et al. 2018); and (vii) they can be combined with traditional antibiotics (Guo et al. 2017).

Endolysins or PG hydrolases constitute a specific group of enzybiotics encoded by bacteriophages and/or prophages in host cells at the end of the bacteriophage lytic cycle (Murray et al. 2021; Love et al. 2020). Previous studies have proven that endolysins exhibit lytic activity against the majority of resistant bacterial cells (Loeffler et al. 2003; Harhala et al. 2018; Abdelkader et al. 2019; Dams and Briers 2019; Premetis et al. 2023a, b, c). Moreover, evaluation tests of endolysins have confirmed their non-toxic behavior after their administration in animal models (Rodríguez-Rubio et al. 2013; Hermoso et al. 2003; Kashani et al. 2018; Guo et al. 2017). Endolysins are classified into amidases, glycosidases, and peptidases, depending on the reactions that catalyze (Szweda et al. 2012). In particular, amidases hydrolyze the amide bond between *N*-acetylmuramic acid (NAM) and an L-amino acid (preferably L-alanine) in the bacterial PG and are further divided into three groups (type 2, type 3, type 5) based on their contained catalytic domain (Vollmer et al. 2008). Glycosidases catalyze the hydrolysis of the glycosidic bond in various oligosaccharides (Höltje 1995), while peptidases cleave peptide bonds between PG amino acids (Höltje 1995).

Hot springs comprise significant ecosystems that have the potential of hosting plentiful and diversified microbial communities. Hence, they could be termed as the “tropical rainforests” of the microbial realm (Des Marais and Walter 2019). Metagenomics enable scientists to obtain genetic resources for novel proteins, enzymes, and metabolites from organisms that are not cultivable, allowing the direct assessment of huge genetic diversity within natural microbial communities, leading to the discovery of novel genes, genomes, and microbial functions (Premetis et al. 2023c). Extremophiles, which are naturally occurring in “extreme” ecological niches, generate thermostable enzymes with economic significance that are useful for bioprocesses and product development (Goh et al. 2022; Swift et al. 2019). These enzymes are quite intriguing for industrial operations since, compared to their mesophilic homologs, they are not only more thermostable but also more resistant to chemical degradation and denaturation. Enzymes that are operationally stable at high temperatures are necessary for many bioprocesses, since they enable benefits such as increased catalytic activity, simple mixing, improved substrate solubility, rapid mass transfer, and less contamination risk (Goh et al. 2022).

Herein, the antimicrobial potential against both gram-negative and gram-positive bacteria of a newly prophage-derived endolysin was investigated. The encoding gene was isolated from a soil environmental sample collected from the thermal springs of Lesvos island, Greece. To the best of our knowledge, this is the first report on the antibacterial activity of this amidase.

## Materials and methods

### Materials

#### Bacterial strains

*Escherichia coli* strain BL21 Shuffle^®^ T7 Express was applied as expression host for the production of NALAA-2. The bacterial strains utilized for the experimental determinations of the amidase lytic activity consisted of the commercially available microorganisms *Micrococcus lysodeikticus* ATCC™ 4698, *Staphylococcus aureus* (MSSA) ATCC™ 25923, and *Enterococcus faecalis* ATCC™ 29212 (Microbiologics SCA). The rest of the bacterial strains examined were cell cultures of the commercial strain *E. coli* DH5a and clinical isolates of *Staphylococcus epidermidis*, *Streptococcus* pyogenes, *Enterococcus faecium*, *Bacillus cereus*, *Acinetobacter baumannii*, and *Clostridium difficile* that were kindly offered by the Department of Microbiology, “Agia Sophia” Children’s Hospital, Athens.

#### Chemicals

The antibiotic kanamycin was purchased from AppliChem (Darmstadt, Germany). Ciproflaxin and gentamicin antimicrobial susceptibility discs were bought from Thermo Fisher Scientific (MA, USA). The Bacterial Viability and Gram Stain kit was obtained from Biotium. SYPRO^®^ Orange Protein Gel Stain was purchased from Sigma-Aldrich, USA (Merck).

### Methods

#### Bioinformatic and structural analysis of Ami1

Crystal structures and protein models were inspected using the program UCSF Chimera 1.16. The amidase sequence was analyzed for the detection of functional regions through InterPro (Blum et al. 2021). The presence of signal peptides was assessed using SignalP 6.0 (Teufel et al. 2022). The structure of the new amidase was predicted through the online server I-TASSER (Yang and Zhang 2015; Zhang et al. 2017). I-TASSER generated five models for amidase and the one with the highest fidelity index (*C*-score =  − 3.91) was used. The structure of amidase was also predicted by AlphaFold (Jumper et al. 2021). The Protein Data Bank (PDB) was used for mining sequences homologous to the Ami1 by employing BLASTp. The obtained sequences were aligned using the Clustal Omega (Madeira et al. 2019; Sievers et al. 2011), and the results were visualized via ESPript (Robert and Gouet 2014). Construction of a phylogenetic tree was achieved through Geneious and the online tool iTOL 5 (Letunic and Bork 2021).

#### Sample collection and DNA extraction

Lesvos is located in the North Aegean Sea on the Aegean-Anatolian microplate between the converging African and Eurasian plates (Pe-Piper et al. 2019). Intense volcanic activity occurred between 18.5 and 17 Ma (Pe-Piper and Piper 1992), endowing a significant number of thermal springs. Wet soil samples from a hot spring in Polichnitos area (39°04′24.8″ N, 26°12′00.9″ E) were collected aseptically in sterile tubes in May 2019. The in situ temperature measurements reached 74 °C. The samples were stored at 4 °C upon return to the laboratory, and subsequent total DNA extraction was accomplished with a DNeasy^®^ PowerMax^®^ Soil kit (QIAGEN, Germany) using 10 g of each sample. Environmental DNA (eDNA) extraction was performed according to the manufacturer’s instructions. The extracted eDNA was concentrated by precipitation with NaCl/cold ethanol, and the resulting pellet was washed with ethanol solution (70% v/v) according to the manufacturer’s instructions. The quality of the purified eDNA was evaluated via agarose gel electrophoresis and quantity by measurements with a NanoDrop^®^ ND-1000 spectrophotometer (Thermo Fisher Scientific Inc., MA, USA). The final yield was approximately 20 ± 7 ng/μL (average ± SD).

#### Amplification of 16S rRNA for high-throughput sequencing

A ~ 460 bp fragment corresponding to the hypervariable region V3–V4 of the 16S rRNA gene was amplified using a specific bacterial primer set consisting of 341F (5′-CCTACGGGNGGCWGCAG-3′) (Herlemann et al. 2011; Klindworth et al. 2013) and 805 Rmod (5′-GACTACNVGGGTWTCTAATCC-3′) (Apprill et al. 2015) primers. Both oligonucleotides carry overhanging Illumina adapters, and total genomic DNA was set as a template to target the specific region. The PCR amplification mixture, the reaction conditions, the 16S Metagenomic Sequencing Library Preparation (Illumina), and the sequencing on an Illumina® MiSeq (PE300) platform were accomplished as described by Salmaso et al. (2018). The amplified sequences were allocated according to sample-specific barcodes and saved in FASTQ-formatted files. Sequencing data were deposited in National Center for Biotechnology Information (NCBI) and can be accessed via BioProject ID PRJNA997949.

#### Bioinformatic analysis

The processing of the raw data incorporated in FASTQ files was achieved through the QIIME 2™ microbiome analysis package, version 2019.4 (Bolyen et al. 2019). Initial paired-end reads corresponding to different samples were merged in a single file following quality control with DADA2 (Callahan et al. 2016). Forward and reverse primers as well as low quality regions of the sequences and chimeric sequences were removed. The exact non-chimeric sequences [amplicon sequence variants (ASVs)] were classified using SILVA 132 reference sequence alignment and majority-consensus taxonomy (seven levels) clustered at 99% sequence similarity. The training of the classifier was carried out using the Naive Bayes algorithm (Pedregosa et al. 2011; Bokulich et al. 2018) based on the multiple alignment of the SILVA 132 database (Quast et al. 2012; Yilmaz et al. 2014). Further downstream analysis included the exclusion of sequences belonging to Archaea, chloroplasts, and mitochondria. Following evaluation of the sequencing depth, a total number of 208 classified ASVs was rarefied to 21,793 sequences (Weiss et al. 2017).

#### Isolation of Ami1 gene and molecular cloning

Gene-specific primers for a potential extremophilic *N*-acetylmuramoyl-L-alanine amidase were designed according to the final classification results of the thermal springs’ eDNA samples targeting for an amidase originally coming from the bacterium *Thermotalea metallivorans*. The sequences of the primers are as follows: (forward) 5′-AGAAGGAGATATACCATGAAGCTTTTTACTAAATACATGAC-3′ and (reverse) 5′-GTGGTGGTGGTGGTGTTCCGGCAATTTGAGAACTTG-3′. The first fifteen nucleotides of each primer correspond to regions of the expressional vector pET-28b(+) while the rest of the sequence is gene specific. The amplification of the gene was achieved via gradient PCR protocol utilizing KAPA Taq DNA polymerase and total genomic eDNA as template for each reaction. The PCR reaction mixture (total volume at 25 μL) consisted of the following components: 1.5 ng eDNA from thermal spring as template, 7.5 pmol of each primer, 50 μM of each dNTP, 1 × KAPA Taq buffer, and 0.5 U KAPA Taq DNA polymerase. The main conditions applied for the reaction were based on the manufacturer’s instructions and included a set of 10 cycles with annealing temperature at 50 °C for 30 s and extension at 72 °C for 90 s, a second step of 10 cycles with annealing temperature at 56 °C for 30 s and extension at 72 °C for 90 s and, finally, a step of 18 cycles performing annealing at 64 °C for 30 s and extension at 72 °C for 90 s. The PCR products were evaluated through 1% w/v agarose gel electrophoresis, and the corresponding amplification fragment was cut off and purified with the NucleoSpin^®^ Gel and PCR Clean-up kit (Macherey-Nagel, Germany), according to the manufacturer’s instructions.

The purified DNA fragment was subsequently inserted to the pGEM^®^-T easy vector (Promega, WI, USA) using a 3/1 insert/vector molar ratio, according to the manufacturer’s instructions. The recombinant plasmid was used to transform high-efficiency competent cells (*E. coli* DH5a), and the successful cloning reaction was assessed through blue or white colony screening on indicator plates containing X-Gal and IPTG. Selected colonies were subjected to plasmid isolation and Sanger sequencing. The sequenced gene was subcloned to the expressional vector pET-28b(+) using the In-Fusion^®^ HD Cloning kit (Takara Bio USA, Inc.) in which homologous recombination is possible due to identical flanking sequences between the gene and the cloning vector. The cloning reaction mixture was used to transform *E. coli* Stellar™ competent cells. The recombinant amidase construct carries a C-terminal 6-His affinity tag.

#### Expression and purification of recombinant Ami1

Heterologous expression of recombinant Ami1 was achieved in *E. coli* BL21 Shuffle^®^ T7 Express cells previously modified to harbor the pET-28b(+) plasmid construct. In particular, transformed cells were plated on LB agar medium containing 30 μg/mL kanamycin following an overnight incubation at 37 °C. Distinct colonies were inoculated in LB medium and were allowed to grow in a shaking incubator at 37 °C and 180 rpm for 14–16 h, and, afterwards, a proper amount of cell culture was transferred to 2xYT medium (1.6% w/v tryptone; 1.0% w/v yeast extract; 0.5% w/v sodium chloride, pH 7) containing an increased amount of kanamycin (100 μg/mL) and was allowed to grow at 37 °C until the optical density at 600 nm reached 0.5. Then, protein expression was induced by the addition of 0.5 mM IPTG and the culture was incubated  at 20 °C under reduced agitation (140 rpm) for 16–20 h. Cells were harvested by centrifugation (8500 × g; 10 min), resuspended in lysis buffer (50 mM NaH_2_PO_4_, 300 mM NaCl, 10 mM imidazole, pH 8), and disrupted by sonication (50 W, 60 Hz, 5 cycles of 15s sonication and 30s interval, ice bath).

Purification of recombinant Ami1 was implemented following previously described protocols (Labrou et al. 2001; Georgakis et al. 2020). The cell lysate containing the soluble amidase was initially centrifuged at 16,000 × g for 10 min, followed by an additional centrifugation at 8500 × g for 10 min. The resulting supernatant was loaded on a Ni-IDA-Sepharose affinity absorbent (0.5 mL) previously equilibrated with lysis buffer. Then, the adsorbent was washed with twenty column volumes (CVs) of equilibration buffer, followed by twelve CVs of washing buffer (50 mM NaH_2_PO_4_ containing 300 mM NaCl, pH 8) and by twelve CVs of washing buffer supplemented with glycerol (20% v/v). A final washing step included fourteen CVs of washing buffer (50 mM NaH_2_PO_4_ containing 300 mM NaCl, pH 6.3). Ami1 was eluted by adding two CVs of buffer containing 50 mM NaH_2_PO_4_, 300 mM NaCl and 200 mM imidazole (pH 8) and three CVs of the same buffer with 250 mM imidazole. All purification steps were carried out at 4 °C. Collected fractions were assayed for enzyme activity through turbidimetry, and their purity was evaluated using SDS-PAGE (12% w/v).

#### Differential scanning fluorimetry

Differential scanning fluorimetry (DSF) was performed to determine the thermal stability of the Ami1 on a real-time PCR device (Applied Biosystems^®^ StepOne™, Thermo Fisher Scientific) (Meekins et al. 2015; Murphy et al. 2022). Briefly, the DSF reaction assays (20 μL) were prepared by mixing appropriate amount of purified enzyme (10 μg) in a buffer system (HEPES/NaOH 0.02 M, pH 7) containing 15 × SYPRO™ Orange (Invitrogen, USA). Fluorescence intensity was monitored following a temperature gradient that covered a range between 10 and 98 °C at a constant scan rate of 1 °C/90 s. All measurements were performed in quadruplicates. The obtained data were successfully used to fit the Boltzmann sigmoidal function curve. The melting temperature (*T*_*m*_) was calculated using the computer program GraphPad Prism version 9.3.1 (GraphPad Prism Software, Inc.).

#### Turbidimetric assays

Turbidimetric assays for the determination of the Ami1 lytic activity were carried out using two gram-negative (*A. baumannii* and *E. coli* DH5a) and seven gram-positive (*S. aureus*, *E. faecalis*, *B. cereus*, *C. difficile*, *E. faecium*, *S. epidermidis*, and *M. lysodeikticus*) bacterial species. The protocol was based on a previously described turbidimetric method (Qafary et al. 2021; Kim et al. 2015; Shugar 1952). Briefly, the reaction mixtures contained bacterial cells (OD_600_ 0.5–0.6), 50 μg of purified amidase, and 1 mM ZnCl_2_. Reaction mixtures were incubated at 25 °C for 90 min, and enzyme lytic activity was investigated by recording the decrease of OD_600_ at 10-min intervals.

#### Effect of pH, temperature, and zinc ions on Ami1 activity

The effect of pH on the lytic activity of the Ami1 was assessed via turbidimetric assays, as described above. The following buffer systems were used (0.05 M): CH_3_COONa, pH 4; CH_3_COONa, pH 5; MES, pH 6; MES, pH 6.5; HEPES, pH 7; HEPES, pH 7.5; Tris-HCl, pH 8; Tris-HCl, pH 8.5; Tris-HCl, pH 9; Glycine-NaOH, pH 9.5; Glycine-NaOH, pH 10; Glycine-NaOH, pH 10.5. Each buffer comprised of suspensions of thermally inactivated *M. lysodeikticus* or *E. coli* DH5a.

To assess the role of Zn^2+^ in enzymatic activity, further turbidimetric experiments were performed. Briefly, appropriate volumes of 0.1 M ZnCl_2_ solution were added to reaction mixtures containing 50 μg of purified Ami1 and bacterial cells (*M. lysodeikticus* or *E. coli* DH5a) to a final concentration ranging from 0.1 to 2 mM. Ami1 lytic activity was investigated by recording the decrease of OD_600_ at 10-min intervals for 90 min. Ami1 reaction mixture containing 1 mM EDTA was used as control.

#### PG isolation

PG isolation from *E. coli* DH5a, *S. aureus*, and *S. epidermidis* was achieved as reported previously (Fukushima and Sekiguchi 2016). The isolated PGs were lyophilized and stored at − 20 °C.

#### Antimicrobial activity assessment through disk diffusion method

The Kirby-Bauer disk diffusion method was used for the determination of the minimum inhibitory concentration (MIC) of Ami1 against *E. coli* DH5a, *S. aureus*, and *S. epidermidis* living cells (Hudzicki 2009). MIC is defined as the lowest concentration of an antimicrobial agent (μg/mL), able to completely inhibit the growth of a test strain (Kowalska-Krochmal and Dudek-Wicher 2021). Overnight bacterial cultures were utilized to prepare suspensions with turbidity values equal to a 0.5 McFarland standard. The suspensions were uniformly spread, under sterile conditions, on Petri dishes containing Mueller-Hinton (MH) agar growth medium (ThermoScientific™ OXOID™). Filter paper discs with different amounts of Ami1 (typically 5–200 µg) were placed on the surface of the plates. Plates were incubated for 24 h at 37 °C prior to evaluating the results.

#### Broth dilution method for the determination of bactericidal activity

The bactericidal activity of Ami1 was examined via the broth dilution method, and the half-maximal lethal concentration (LC_50_) and the minimum bactericidal concentration (MBC) were determined. Cultures of three different bacterial strains (*E. coli* DH5a, *S. aureus*, and *S. epidermidis*) at their stationary phase of growth were appropriately diluted in Luria-Bertani (LB) medium to OD_600_ corresponding to a 0.5 McFarland turbidity standard. Further dilutions were performed to prepare suspensions with bacterial concentrations of approximately 10^5^ CFU/mL. Equal amounts of each bacterial suspension were mixed with ZnCl_2_ (1 mM) and different amounts of Ami1 (0 μg, 25 μg, 50 μg, 100 μg, 125 μg, 150 μg, and 200 μg) to 1 mL final volume. Following 1.30h, 3h, and 6h incubation at room temperature, 10 μL of each enzyme reaction was spread on LB agar plates and incubated at 37 °C for 24 h.

## Results

### An overview at the microbial communities of the soil sample from a hot spring of Polichnitos

**Fig. 1:**
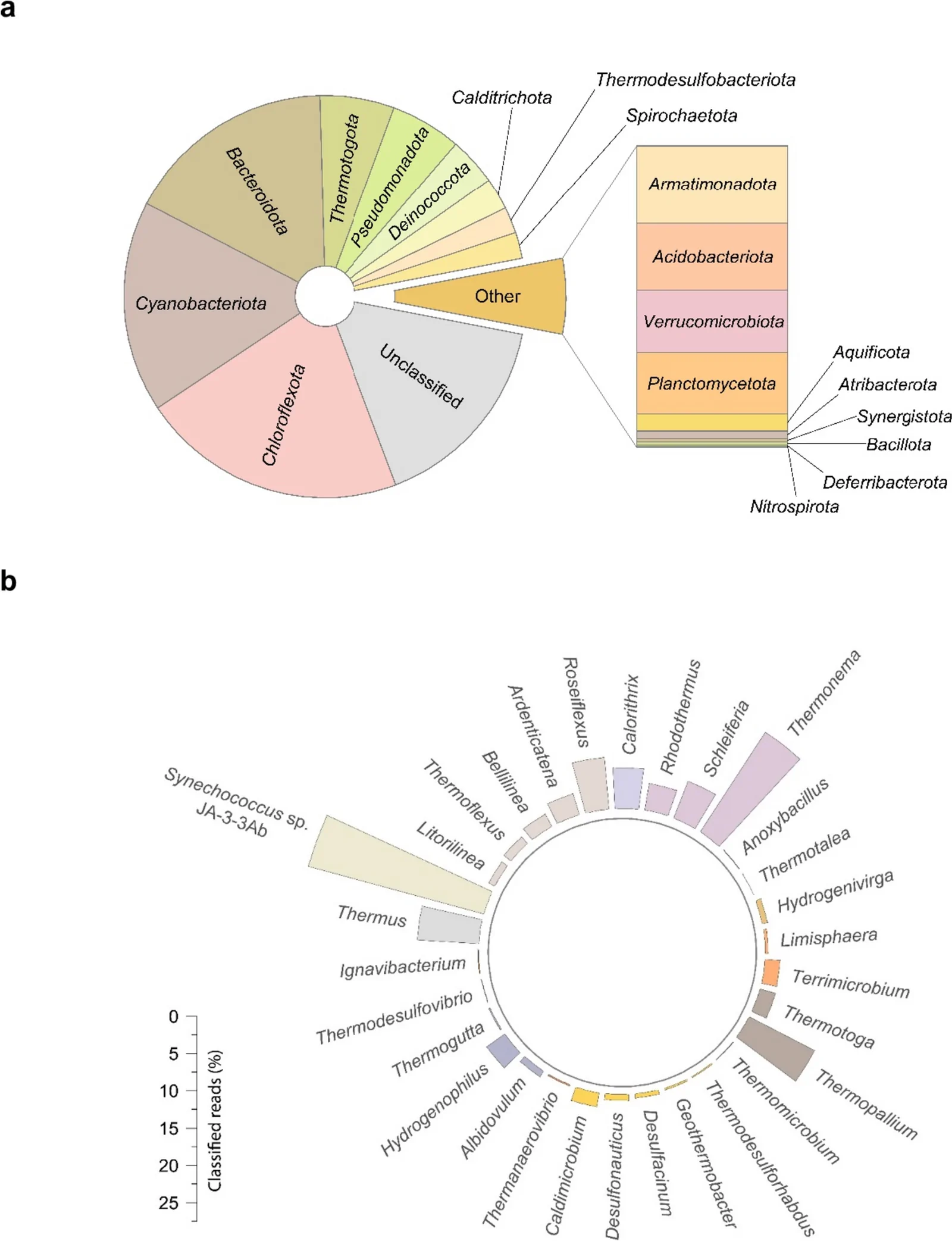
**a** Representative Phyla of the microbial communities of the hot spring of Polichnitos, Lesvos (Greece), following classification of the V3–V4 hypervariable region of the 16S rRNA gene. **b** Representative thermophilic bacterial genera that were identified through the analysis of the bacterial communities of the hot spring. The depicted genera correspond to approximately 45% of the total reads initially acquired during the sequencing of the V3–V4 hypervariable region of the 16S rRNA gene

In the present work, 16S rRNA gene analysis of soil samples from a hot spring of Polichnitos allowed the classification of the amplified V3–V4 fragments. The analysis revealed 192 ASVs, belonging to bacteria. Among all ASVs, 39 were grouped as “unclassified” having been identified only to Domain/Kingdom level or were downgraded to unclassified due to reclassification changes, corresponding to 16% of the total sample reads (Oren and Garrity 2021; Oren et al. 2022; de Vienne 2016). The most abundant representative Phyla of the microbial community of the hot spring of Polichnitos are shown in Fig. 1a. Figure 1b depicts representative thermophilic bacterial genera that were identified. A total of 19 Phyla appears to comprise the microbial population of the sample site.

### Bioinformatics and in silico characterization of a new endolysin from thermophilic genera

A manual BLASTn search of these classified sequences led to the identification of a small group of thermophilic genera (Fig. 1b). Among them, the strictly anaerobic and thermophilic genus *Thermotalea* that belongs to the family of *Clostridiaceae* (Phylum Bacillota) is less characterized, and so far, only one known species (*Thermotalea metallivorans*) has been isolated and characterized (de Vienne 2016; Ogg and Patel 2009). Genome search on the available *Thermotalea metallivorans* genomic dataset (BioProject accession: PRJNA294866, ID 294866) revealed an open reading frame (ORF) (Protein ID: WP_068556374.1), encoding an endolysin that belongs to the cell wall amidase type 2 (NALAA-2) (Vermassen et al. 2019). Endolysins, which belong to the NALAA-2 family, have attracted significant attention because of their enhanced antimicrobial activity (Broendum et al. 2018; Vermassen et al. 2019; Premetis et al. 2023b). We selected to analyze endolysins since they have received much interest from the scientific community as potential powerful antimicrobials in the prophylaxis and treatment of bacterial infections due to their specificity, mode of action, potential for engineering, and lack of resistance mechanisms. Enzymes from thermophile organisms are not only more thermostable, but also more resistant to chemical degradation and denaturation than their mesophilic homologues, making them extremely useful tools in biotechnology (Swift et al. 2019; Love et al. 2021; Goh et al. 2022).

PCR using primers specific to the WP_068556374.1 resulted in the amplification of a nucleotide sequence encoding an NALAA-2 similar, but not identical, to the WP_068556374.1. The PCR amplified amidase sequence shares 97.9% sequence similarity with the enzyme encoded by *T. metallivorans* (WP_068556374.1), while the highest sequence similarity was observed with the NALAA-2 (Accession No. WP_103082047.1; SeqID 98.8%), from another thermophilic species, namely *Pseudoclostridium thermosuccinogenes* (Phylum Bacillota, Family *Oscillospiraceae*).

The available genome of *P. thermosuccinogenes* (GenBank: CP021850.1) was investigated with the aim of detecting whether the NALAA-2 gene in the bacterial chromosome has prophagic origin. The web tools PHASTER (Arndt et al. 2016; Zhou et al. 2011) and Prophage Hunter (Song et al. 2019) allowed the identification of a prophage region within a bacterial genome as well as to annotate the encoded ORFs. Both searches within *T. thermosuccinogenes* genome revealed the existence of a prophage region (Prophage Hunter: location region bp 3050934–3096389; PHASTER: location region bp 3047743–3096668) that includes a NALAA-2 gene (genome location region bp 3053564–3054568; GeneBank: AUS97328.1, identical to WP_103082047.1), annotated as a hypothetical protein by PHASTER and as an amidase by Phage Hunter. The latter predicted the candidate prophage area with a high score (0.88), indicating an active phage region (Song et al. 2019). The high sequence similarity of the newly identified amidase (denoted Ami1) with the prophage amidase from *T. thermosuccinogenes* points to the conclusion that Ami1 is a prophage-encoded enzyme (endolysin). The Ami1 gene sequence was deposited at the GenBank under the accession number OQ725913.

Ami1 consists of 334 amino acids, and its theoretical molecular mass and isoelectric point are predicted 38.72 kDa and 9.07, respectively. InterPro analysis (Blum et al. 2021) of Ami1 sequence revealed the existence of two distinct structural domains, an NALAA-2 domain and a LysM. The former domain (IPR002502) is located between positions 8–169 and belongs to the superfamily of homologous proteins classified as PG recognition proteins (PGRPs) (IPR036505, cd06583). The LysM region, one of the most common cell wall binding domains (CWBDs) that can be found in endolysins, is located between positions 286–333 (IPR018392). The two domains are connected by a sequence (linker) without any known functional feature. The NALAA-2 enzymes are Zn^2+^-dependent (E.C. 3.5.1.28) and catalyze the hydrolysis of the amide bond between *N*-acetylmuramic acid (NAM) and an L-amino acid (preferably L-Ala) in the PG structure (Vermassen et al. 2019; Lee et al. 2013; Martínez-Caballero et al. 2013; Pennartz et al. 2009). Analysis of Ami1 for the presence of signal peptides (SignalP-6.0, Teufel et al. 2022) or SAR domains (Oliveira et al. 2013; Gontijo et al. 2022) revealed the absence of all known types of SP/SAR domains. Ami1 sequence does not fulfil the criteria for a SAR domain (e.g., transmembrane domain located at the N-terminus with a glycine and alanine content of 40 to 60% and 0 to 2 basic residues, usually Lys).

**Fig. 2:**
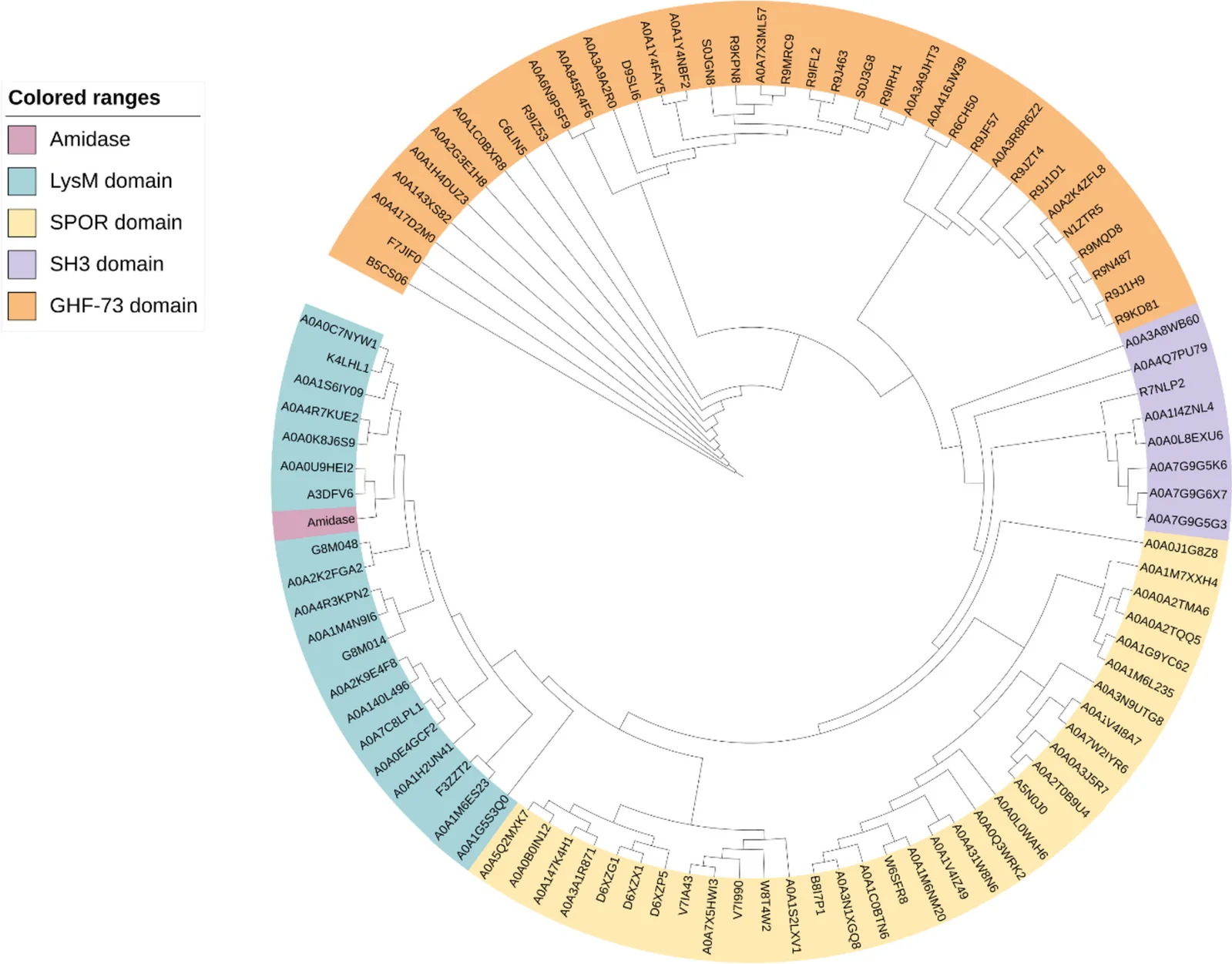
Phylogenetic analysis of the Ami1. The Ami1 sequence with representative homologous NALAA-2 of phage or prophage origin that possess different CWBD (LysM, SPOR, SH3, or GHF-73) commonly found in amidase family were used for phylogenetic analysis. Geneious was used for tree construction and illustrated using iTOL v5 (Letunic and Bork 2021). Sequences with the LysM domain are shown in blue color. Sequences with the SPOR domain are in yellow. Sequences with the SH3 domain are in purple. Sequences with the GHF-73 are in orange. The Ami1 sequence is shown in dark pink. LysM, lysin motif; SPOR, Sporulation-related repeat; SH3, Sarcoma homology-3; GHF-73, glycosyl hydolase family 73

Phylogenetic analysis of Ami1 sequence with representative homologous NALAA-2 of phage or prophage origin that possess different types of CWBD (LysM, SPOR, SH3 or GHF-73), commonly found in amidase family (Vermassen et al. 2019), was carried out, and the results are depicted in Fig. 2. The results showed that sequences are clustered into four distinct clades, based on the presence of the CWBD (LysM, SPOR, SH3, or GHF-73). Clearly, the Ami1 sequence is clustered together with the amidase sequences that possess the LysM domain (Buist et al. 2008; Yahashiri et al. 2015, 2017; Vermassen et al. 2019; Whisstock and Lesk 1999; Albrecht et al. 2012).

**Fig. 3:**
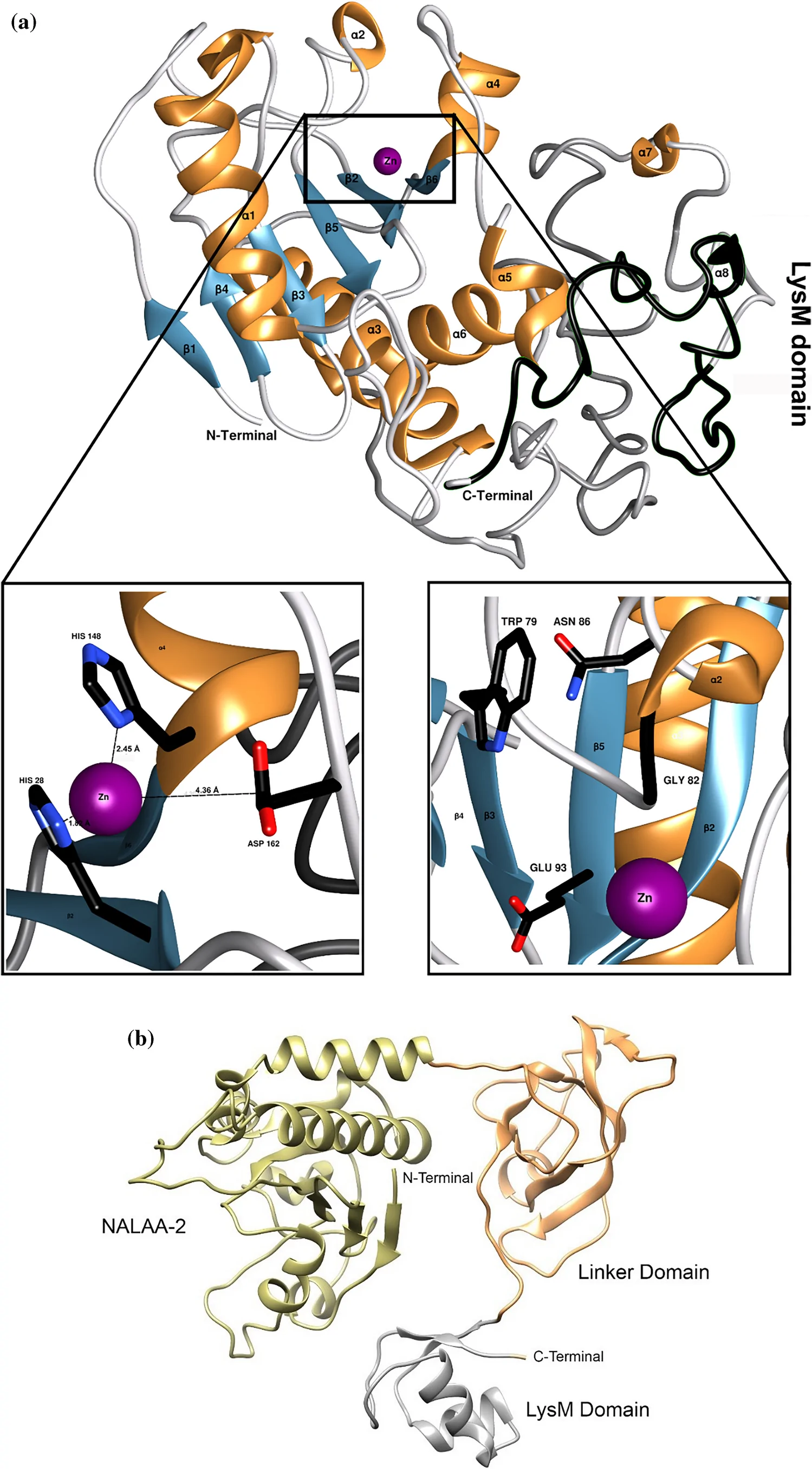
**a** The predicted structure of Ami1 by iTASSER (Yang and Zhang 2015). Overview of the Ami1 structure in complex with Zn^2+^. The LysM domain at the C-terminus of the structure is shown in black. Secondary structure elements are labeled. The figure was created using the program UCSF Chimera 1.16. Left zoom: predicted Zn^2+^ binding residues: His28, His148, and Asp162. Right zoom: key conserved amino acid residues (Trp79, Gly82, Asn86, and Glu93), predicted to contribute in PG binding and/or catalysis. **b** The structure of Ami1 as predicted by AlphaFold (Jumper et al. 2021). The NALAA-2 and LysM domains are colored green and grey, respectively. The linker domain is colored orange and labeled

Molecular modeling and structure prediction were implemented using the I-TASSER server (Yang and Zhang 2015; Zhang et al. 2017). I-TASSER generated five models, and the one with the highest fidelity index was selected for further investigation (Fig. 3a). Ami1 is predicted to consist of six β-strands that form a β-sheet structure inside the protein molecule, as well as of four large α-helices (α1, α3, α4, and α6) and four smaller ones (α2, α5, α7, and α8). The LysM region was modeled (Fig. 3) at the C-terminus of the amidase domain. This region comprises of 45 amino acids, and its predicted secondary structure includes one short α-helix (α8) at the position 306–308. The Ami1 structure was also predicted using AlphaFold (Jumper et al. 2021; Mirdita et al. 2022) (Fig. 3b). In the AlphaFold structure, the NALAA-2 domain adopts similar overall fold (see Figure S1), consisted of a β-sheet, formed by six β-strands, as well as of four large and five smaller α-helices. The main difference between these two structures is observed in the LysM domain. AlphaFold predicted a structure that consisted of two β-strands and two α-helices (α12 and α13). These differences may be due to the different methodology and algorithms that are used by I-TASSER (Yang and Zhang 2015; Zhang et al. 2017) and Alphafold (Jumper et al. 2021).

**Fig. 4:**
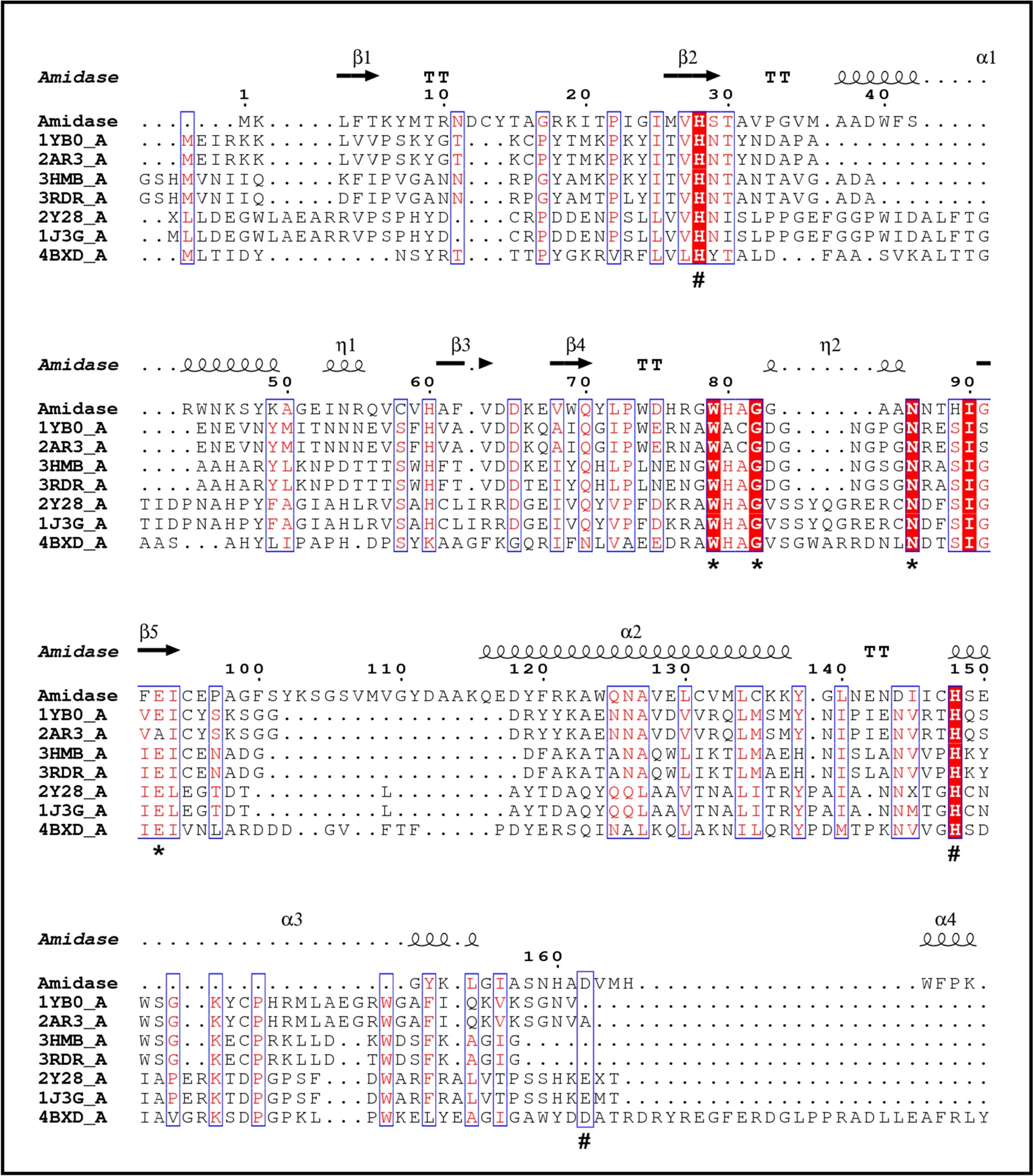
Multiple sequence alignments of Ami1 with homologous endolysins with characterized crystallographic structure and function. Alignment was performed using ClustalΟ and displayed using ESPript 3.0. The sequences used were the following: WP_000405801.1 (1YB0_A, *Bacillus anthracis* SeqID 29.59%), WP_000405801.1 (2AR3_A, *Bacillus anthracis* SeqID 28.57%), WP_148342977 (3HMB_A, *Bacillus subtilis* SeqID 33.70%), WP_071580572.1 (3RDR_A, *Bacillus subtilis* SeqID 30.15%), WP_003018719 (2Y28_A, *Citrobacter freundii* SeqID 28.89%), WP_047715961.1 (1J3G_A, *Citrobacter freundii* SeqID 24.82%), and WP_003163244.1 (4BXD_A, *Pseudomonas aeruginosa* SeqID 29.73%). Conserved areas are shown shaded. A column is framed, if more than 70% of its residues are similar according to physico-chemical properties. The predicted active site residues are labeled with stars and the Zn-binding residues with hash marks

Multiple sequence alignments of Ami1 with homologous endolysins with well characterized structure and function (homology 24.82–33.70%, Fig. 4) allowed the identification of the conserved His28, His148, and Asp162 as residues involved in Zn^2+^ binding, in agreement with those reported in other members of the NALAA-2 family (Low et al. 2005; Kim et al. 2003; Wang et al. 2003; Cheng et al. 1994; Guan et al. 2004; Lim et al. 2006; Leone et al. 2008). Analysis of the predicted I-TASSER structure using COFACTOR (Roy et al. 2012) led to the identification of important residues that are involved in the formation of the binding catalytic site: Tpr79, Gly82, Asn86, and Glu93 (Fig. 3a). Amino acid sequence alignments (Fig. 4) confirmed that Tpr79, Gly82, Asn86, and Glu93 are conserved, despite the low overall sequence homology shared by the enzymes, suggesting that these residues may play important catalytic and/or structural roles (Liepinsh et al. 2003; Carrasco-López et al. 2011; Low et al. 2005, 2011; Büttner et al. 2014; Lee et al. 2013; Zoll et al. 2010).

#### Heterologous expression, purification, and thermostability assessment of Ami1

**Fig. 5:**
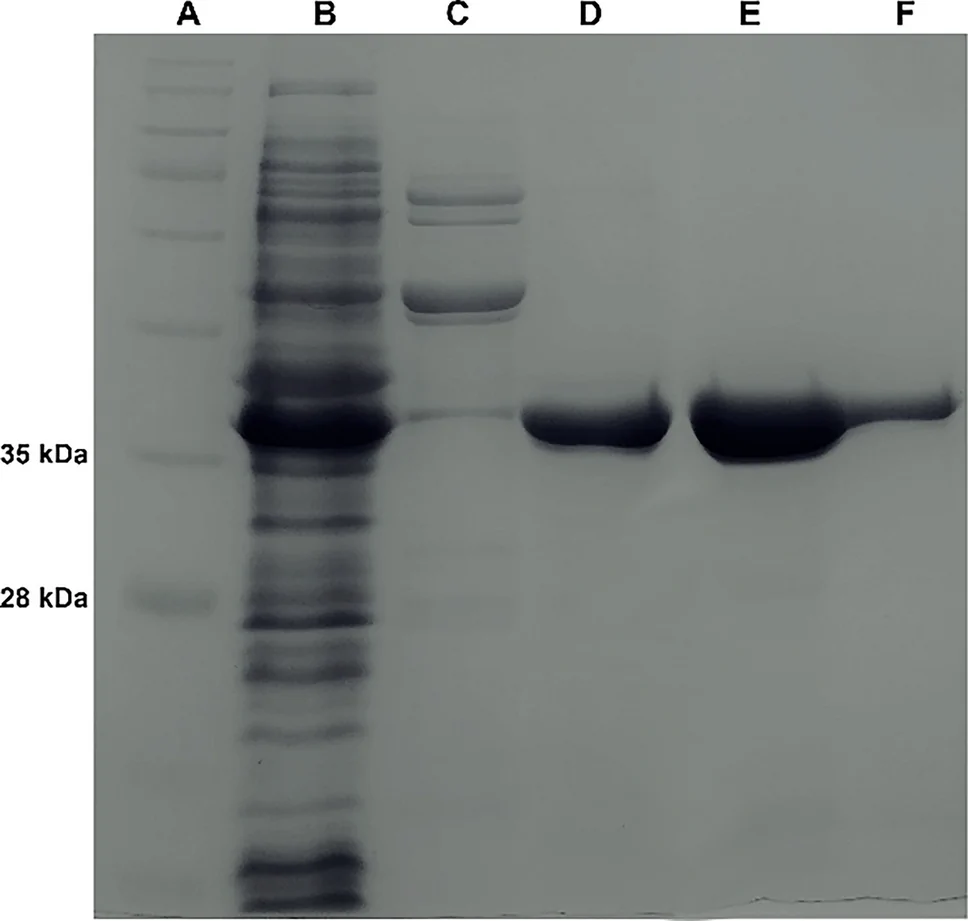
SDS-PAGE (12% w/v) analysis of Ami1 purification by affinity chromatography on Ni^2+^-IDA-Sepharose. A: protein ladder. B: crude extract from *E. coli* BL21 Shuffle^®^ T7 Express. C: eluted fraction with 0.05 M imidazole, D: eluted fraction with 0.1 M imidazole, E: eluted fraction with 0.15 M imidazole, and F: eluted fraction with 0.2 M imidazole

**Fig. 6:**
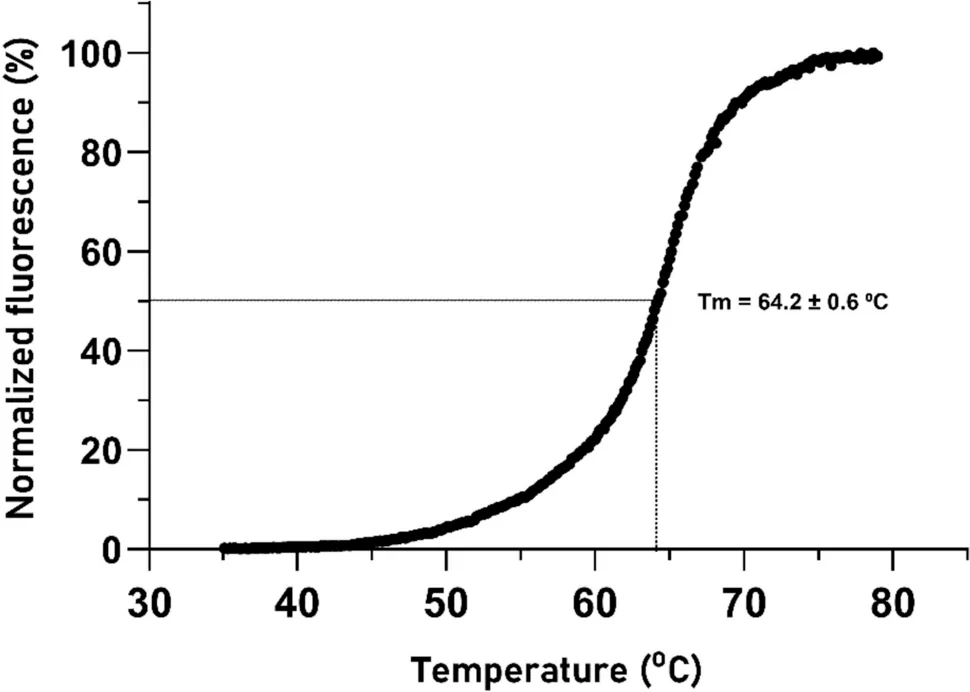
Thermal stability of Ami1. DSF curve of Ami1 for the determination of melting temperature (*T*_*m*_) in 20 mM HEPES buffer, pH 7

The gene of Ami1 was cloned into the pET-28b(+) expression plasmid and used for protein expression in *E. coli* BL21 Shuffle^®^ T7 Express cells. Ami1 was expressed as a 6-His tagged protein for facile and efficient purification using affinity chromatography on Ni^2+^-IDA-Sepharose adsorbent (Fig. 5). The thermal stability of Ami1 was evaluated in 0.02 M, HEPES/NaOH buffer, pH 7, using differential scanning fluorimetry (DSF), and the results are shown in Fig. 6. The melting temperature (*T*_*m*_) of Ami1 that corresponds to the temperature at which 50% of the protein is unfolded was estimated at 64.2 ± 0.6 °C.

#### Demonstration of lytic activity against a range of bacterial species

**Fig. 7:**
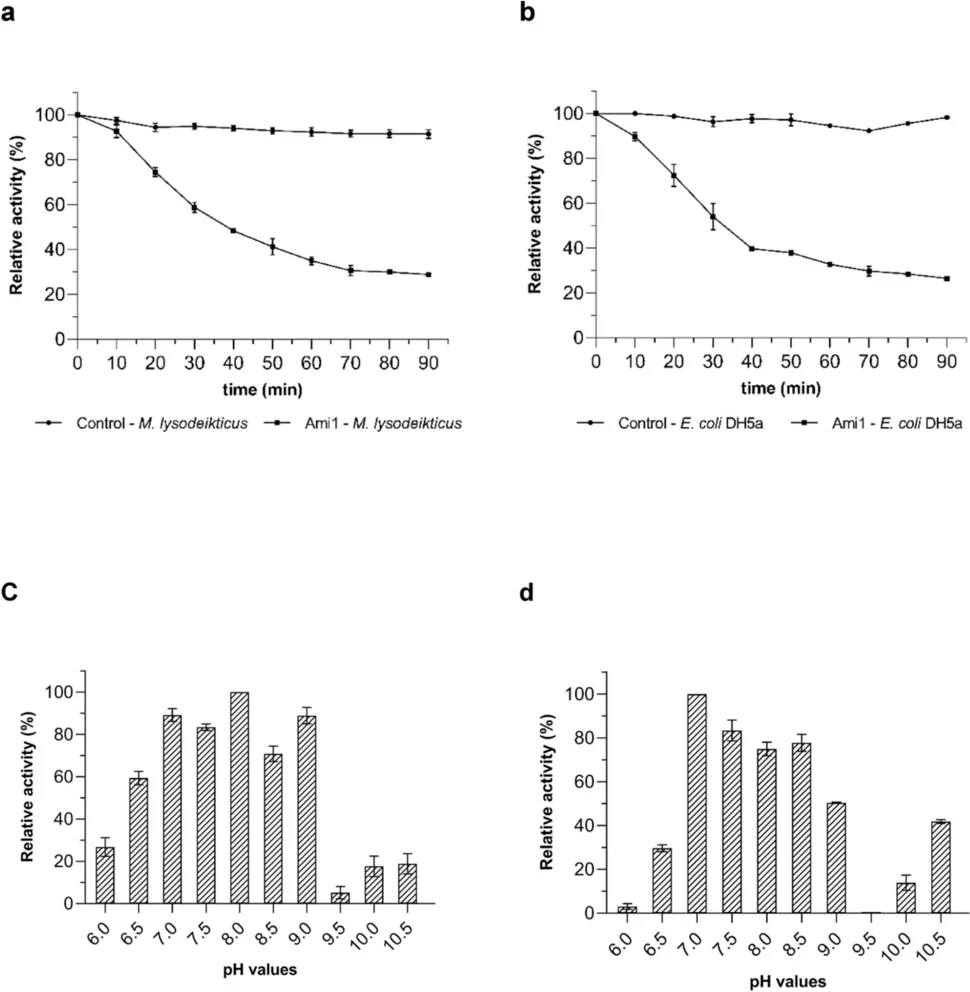
Demonstration of lytic activity using turbidity reduction assays. **a** Lytic activity against *M. lysodeikticus* cells used as substrate. Turbidity reduction was measured spectrophotometrically at 600 nm using *M. lysodeikticus* cells in the absence (control) and presence of the enzyme. **b** Lytic activity against *E. coli DH5a* cells used as substrate. Turbidity reduction was measured spectrophotometrically at 600 nm using *E. coli* DH5a cells in the absence (control) and presence of the enzyme. **c** The effect of pH on lytic activity using *M. lysodeikticus* cells. **d** The effect of pH on lytic activity using *E. coli* DH5a cells. All assays were performed in triplicate. The error bars represent the standard deviation of a dataset

Turbidimetric assays were carried out to demonstrate the lytic activity of Ami1 against model bacterial cells. In the absence of any available information for the enzyme, the assays were carried out at different pH conditions (pH 6–9), employing *M. lysodeikticus* and *E. coli* DH5a as representative gram-positive and negative strains. The results showed that Ami1 exhibits a time-dependent bacteriolytic activity (Fig. 7a, b), being active towards both the gram-positive and negative representative strains. The enzyme exerts its high activity at relatively broad pH range, between 7 and 8. The lytic activity of Ami1, in different pH values, appears to exhibit a strain-dependent profile and reaches the highest value at pH 7 or 8 for *E. coli* DH5a and *M. lysodeikticus* cells, respectively (Fig. 7c, d). Significant decrease in activity was observed above pH 9.0 and below pH 6.5 in both *M. lysodeikticus* and *E. coli* DH5a strains.

**Fig. 8:**
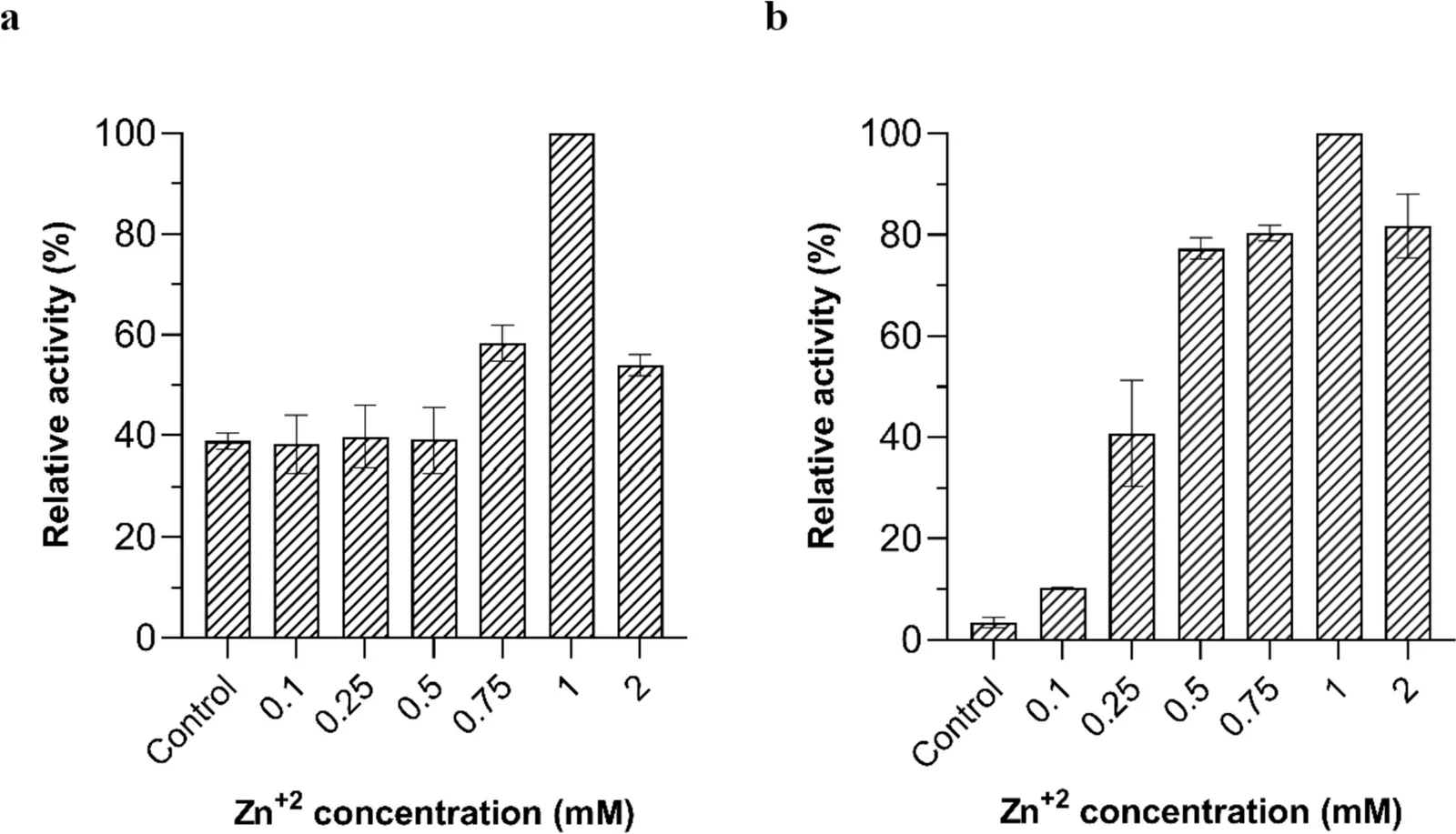
**a** The effect of different concentrations of Zn^2+^ on lytic activity using *M. lysodeikticus* cells. **b** The effect of different concentrations of Zn^2+^ on lytic activity using *E. coli* DH5a cells. The control bar (Fig. 8b) represents the lytic activity of Ami1 after treatment with EDTA (1 mM)

The effect of different Zn^2+^ concentrations on lytic activity was evaluated using *M. lysodeikticus* and *E. coli* DH5a cells. The results (Fig. 8a, b) revealed that the lytic activity increases in a dose-dependent manner in the presence of Zn^2+^ and exhibits its highest activity at 1 mM ZnCl_2_. These results further confirm that Ami1 is a Zn^2+^-dependent enzyme.

**Fig. 9:**
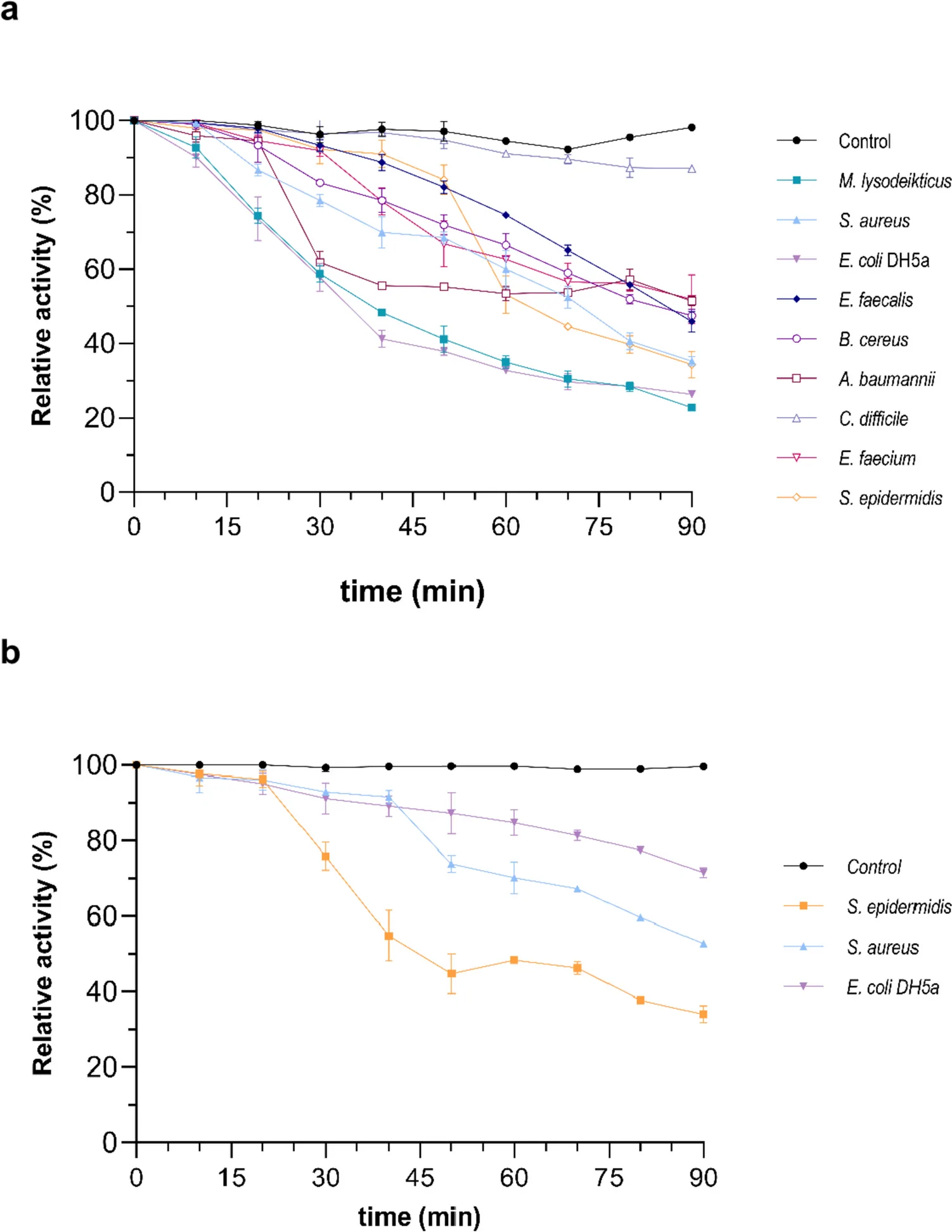
**a** Activity of Ami1 against representative gram-positive and gram-negative bacterial strains. Enzyme activity was demonstrated by measuring the time-dependent turbidity changes of cells suspensions. The results show the relative enzymatic activity (%) at 600 nm after adding Ami1 (50 μg/mL) for 90 min. The relative enzyme activity was calculated as a percentage decrease of the initial OD (600 nm) of the cell suspension. **b** Relative enzymatic activity (%) against PG isolated from *S. epidermidis*, *S. aureus*, and *E. coli* DH5a cells. The assay was carried out for 90 min, and the absorbance was measured at 600 nm. The relative enzyme activity was calculated as a percentage decrease of the initial OD (600 nm) of the PG suspension. In all assays, the percentage decrease of the control reaction (in the absence of enzyme) was subtracted. All assays were performed in triplicate. Error bars represent the estimated standard deviation

In order to assess the selectivity of Ami1 and to identify potential pathogenic or MDR bacterial targets, a variety of different gram-positive and negative bacteria were examined. The results (Fig. 9) demonstrate that Ami1 displays broad lytic specificity. Slight preference was observed against the clinical isolates *S. aureus* and *S. epidermidis* as well as the model strains *M. lysodeikticus* and *E. coli* DH5a. The lowest activity was observed against the *E. faecium* and *C. difficile* cells.

Taking into consideration that *E. coli*, *S. aureus*, and *S. epidermidis* strains are pathogenic and MDR bacteria, they were selected for further studies. To confirm Ami1 functions as PG hydrolase, its lytic activity was assessed using purified PG. Figure 9b illustrates that Ami1 is capable to catalyze the lysis of purified PG isolated from the gram-negative *E. coli* DH5a and the gram-positive *S. aureus* and *S. epidermidis*. The enzyme appears to display higher activity towards PGs from *S. aureus* and *S. epidermidis* (approx. 85%), although its activity towards *E. coli* DH5a PG was significantly lower (58 ± 3%).

#### Effect of Ami1 on live cultures of bacterial cells

**Fig. 10:**
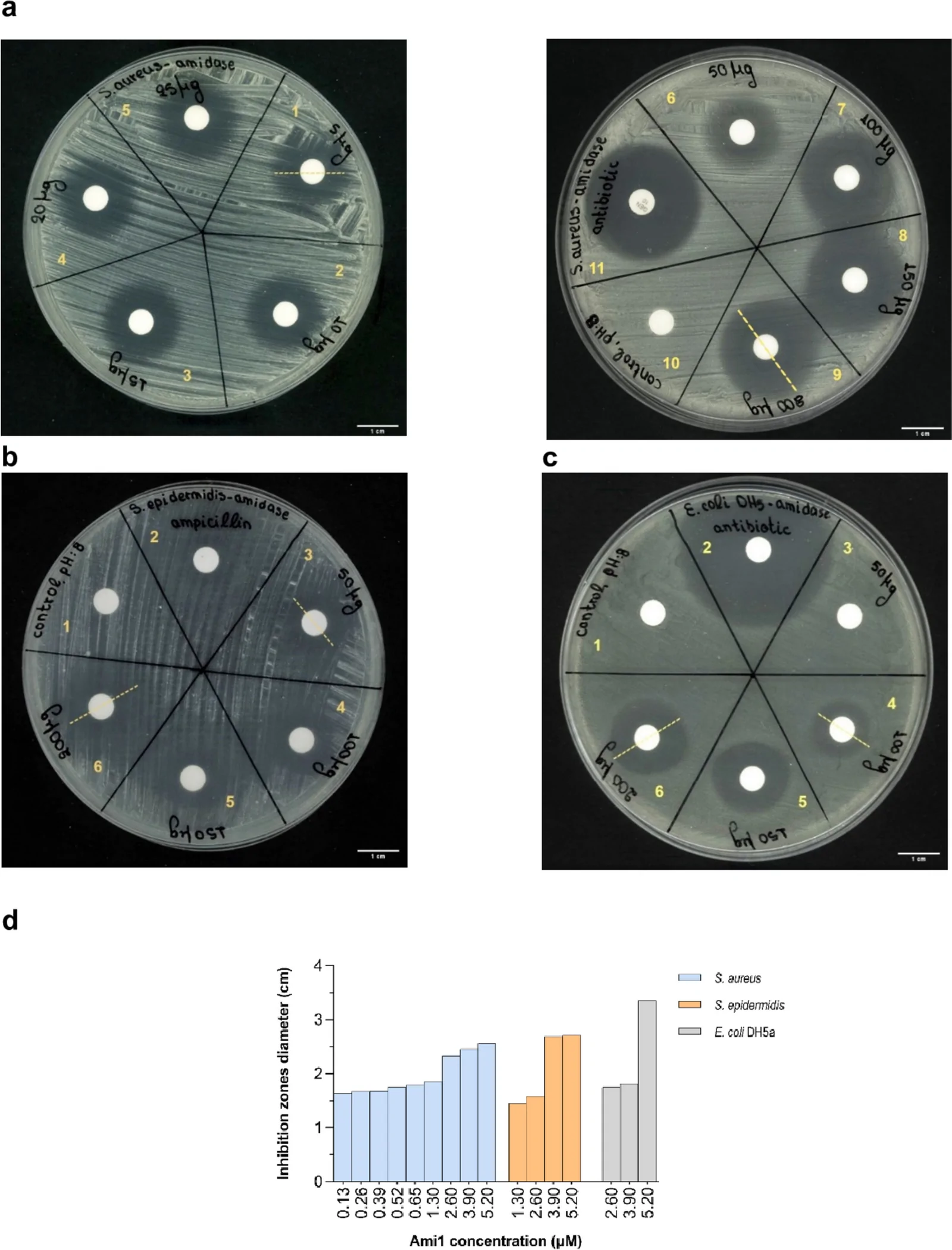
**a** Disk diffusion method against *S. aureus* cells. 1: 0.13 μΜ; 2: 0.26 μΜ; 3: 0.39 μΜ; 4: 0.52 μΜ; 5: 0.65 μΜ; 6: 1.30 μΜ; 7: 2.60 μΜ; 8: 3.90 μΜ; 9: 5.16 μΜ; 10: control (0 μΜ); 11: antibiotic (gentamycin 10 μg). **b** Disk diffusion method against *S. epidermidis* cells. 1: control (0 μΜ); 2: antibiotic (ampicillin 15 μg); 3: 1.30 μΜ; 4: 2.60 μΜ; 5: 3.90 μΜ; 6: 5.16 μΜ. **c** Disk diffusion method against *E. coli* DH5a. 1: control (0 μΜ); 2: antibiotic (ampicillin 15 μg); 3: 1.30 μΜ, 4: 2.60 μΜ; 5: 3.90 μΜ; 6: 5.16 μΜ. **d** Diameter of inhibition zones formed at different Ami1 concentrations (see Fig. 10a–c) for *S. aureus* (blue), *S. epidermidis* (orange), and *E. coli* DH5a (grey) bacteria

To further investigate the antibacterial activity of Ami1, the Kirby-Bauer disk diffusion method (Hudzicki 2009) against live cultures of the target strains was implemented. Figure 10a–c show the effect of different concentrations of purified Ami1 on live cultures on solid agar plates. The results indicated that the growth of bacterial cells was significantly inhibited as the quantity of Ami1 increased, suggesting a dose-response effect. Based on the results (Fig. 10a–c), it is evident that Ami1 exerted the strongest antibacterial effect against *S. aureus.* and *S. epidermidis*. The minimum inhibitory concentration (MIC) of Ami1 against *S. aureus* was determined 0.13 μΜ, as a clear zone of inhibition with a diameter of 1.73 cm was formed. In the case of *S. epidermidis*, the MIC was estimated 1.30 μΜ and the inhibition zone was measured 1.45 cm. At the same concentration, no inhibition of *E. coli* DH5a cell growth was observed; however, significant antimicrobial activity was observed at higher Ami1 concentrations. In particular, at 2.60 μΜ and 5.20 μΜ Ami1, the inhibition zones were 1.36 cm and 1.81 cm, respectively, and the MIC was determined at 2.60 μΜ.

**Fig. 11:**
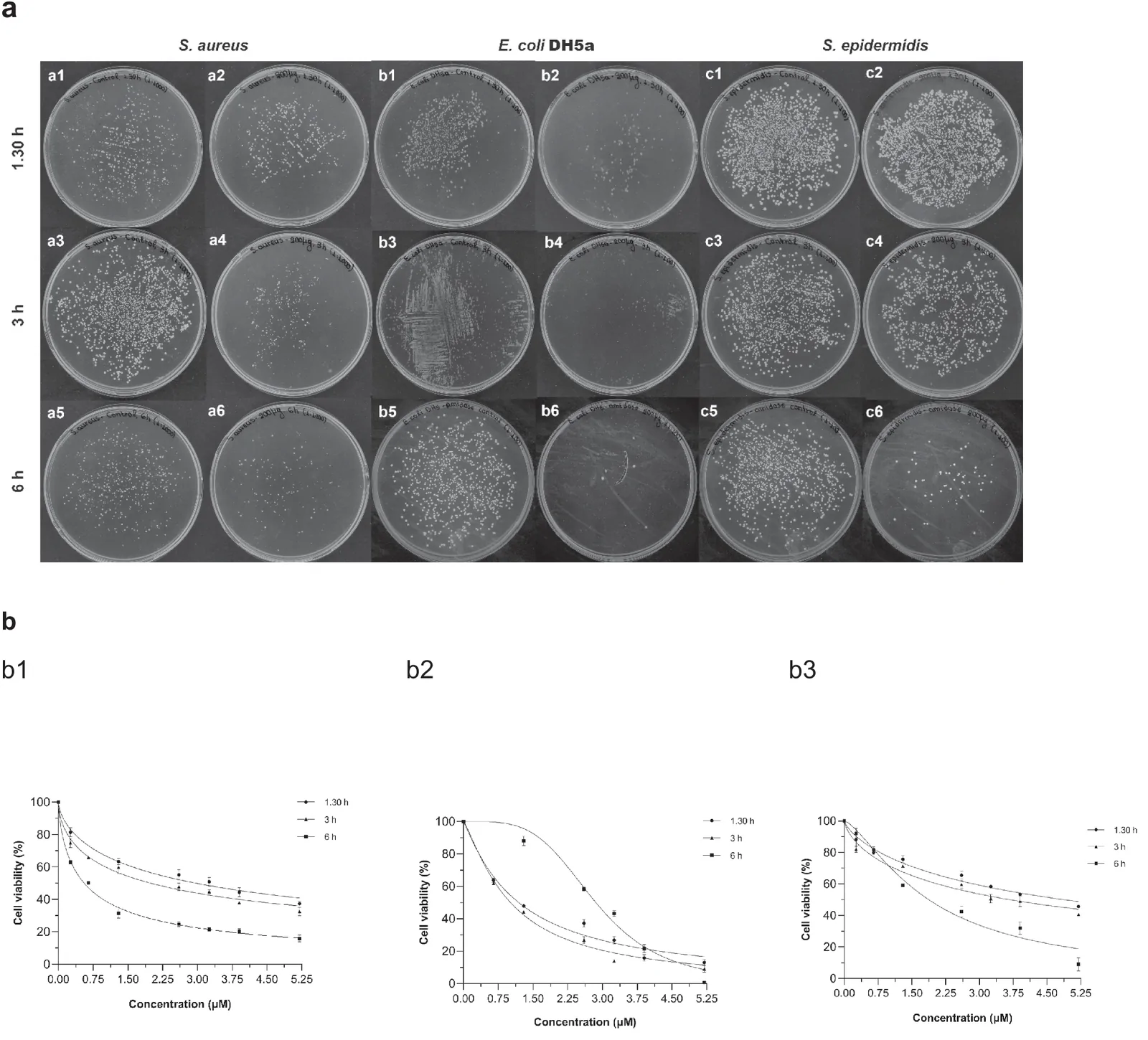
**a** Broth dilution method. a1–a2: 0 μM (control); 5.20 μM of amidase against *S. aureus* after 1.30-h incubation. a3–a4: 0 μM (control); 5.20 μM of amidase against *S. aureus* after 3-h incubation. a5–a6: 0 μM (control); 5.20 μM of amidase against *S. aureus* after 6-h incubation. *E. coli* DH5a cells. b1–b2: 0 μM (control); 5.20 μM of amidase against *E. coli* DH5a cells after 1.30-h incubation. b3–b4: 0 μM (control); 5.20 μM of amidase against *E. coli* DH5a cells after 3-h incubation. b5–b6: 0 μM (control); 5.20 μM of amidase against *E. coli* DH5a cells after 6-h incubation. c1–c2: 0 μM (control); 5.20 μM of amidase against *S. epidermidis* cells after 1.30-h incubation. c3–c4: 0 μM (control); 5.20 μM of amidase against *S. eppidermidis* cells after 3-h incubation. c5–c6: 0 μM (control); 5.20 μM of amidase against *S. eppidermidis* cells after 6-h incubation. **b** Bactericidal effect of different concentrations of Ami1 (see Fig. 11a) on *S. aureus* (b1), *E. coli* DH5a (b2), and *S. epidermidis* (b3) cultures, after 1.30-h, 3-h, and 6-h incubation

Moreover, the bactericidal effect of Ami1 was examined on live bacterial cultures via the broth dilution method. As illustrated in Fig. 11a, b, the number of colonies was gradually decreased in a dose- and time-dependent manner on the three different bacterial species. In particular, incubation of *S. aureus* cells with 5.20 μΜ Ami1 for 1.30 h and 6 h led to 60% and 85% reduction in the number of colonies, respectively [Fig. 11 (b1)]. The bactericidal effect of Ami1 was stronger on *E. coli* and *S. epidermidis* cultures as the incubation of 5.20 μΜ enzyme for 6 h resulted to the elimination of more than 95% of colonies (Fig. 11 (b2, b3)). The acquired results suggest an efficient bactericidal activity and allowed the determination of the minimum bactericidal concentration (MBC) and the half-maximal lethal concentration (LC_50_) of Ami1 against *S. aureus*, *S. epidermidis*, and *E. coli* DH5a, after 1.30 h, 3 h, and 6 h of incubation (Table 1). The bactericidal activity of Ami1 falls within the range expected for highly active endolysins that have been recently reported (Guo et al. 2017; Wu et al. 2018; Plotka et al. 2019; Varotsou et al. 2023; Premetis et al. 2023b).

**Table 1:** MBC (μM) and LC_50_ (μM) values of Ami1 against *S. aureus*, *S. epidermidis*, and *E. coli* DH5a, after 1.30 h, 3 h and 6 h of incubation

Bacterial strains	MBC (μM)	LC_50_ (μM)		
		1.30 h	3 h	6 h
*S. aureus*	0.26 ± 0.05	2.98 ± 0.37	1.99 ± 0.24	0.56 ± 0.06
*S. epidermidis*	0.65 ± 0.05	4.89 ± 0.70	3.68 ± 0.58	1.88 ± 0.30
*E. coli* DH5a	0.65 ± 0.05	1.19 ± 0.18	1.02 ± 0.11	2.86 ± 0.20

## Discussion

The rapid emergence of pathogenic bacterial strains that display resistance to a variety of widely used antibiotics is a growing global threat to public health (Harhala et al. 2021; Laxminarayan et al. 2013; Murray et al. 2021). In the present work, a new prophage encoded endolysin that belongs to the NALAA-2 family was characterized, and its antimicrobial potency against both gram-positive and gram-negative bacteria was investigated. Endolysins encoded by phages constitute a promising class of enzymes with antimicrobial activity, due to their potential to hydrolyze the PG of the bacterial cell wall (Murray et al. 2021; Love et al. 2020; Abdelkader et al. 2019; Dams and Briers 2019).

Ami1 was discovered through mining of soil metagenomic libraries collected from the hot springs of Lesvos Island. Soil from geothermal springs is a challenging natural environment which can give valuable genetic resources, regarding the microbial community size and the diversity of species present including thermophilic and hyperthermophilic microorganisms (Sharp et al. 2014; Lavrentyeva et al. 2018; Kochetkova et al. 2020; Shu and Huang 2022; Peach et al. 2022). Omics technologies such as a marker gene analysis which includes the 16S rRNA gene have revolutionized our insight of microbial diversity and ecology, allowing a better understanding of the microbial communities and their relationships across different taxa, phylogenies, and functional composition in space and time (Goh et al. 2022; Shaffer et al. 2022). A marker gene analysis of an eDNA sample captures a low-resolution picture of a specific microbial community, yet it is an established, quick, and cost-effective method (Knight et al. 2018). The results showed that the microbial population of the sample site comprises a total of 19 Phyla. The relatively restricted microbial diversity of the hot spring of Polichnitos could be attributed to the high temperature of the sampling area (74 °C). Approximately half of the total ASVs belong to the Phyla Chloroflexota, Cyanobacteriota, and Bacteroidota, which seem to be predominant. The remaining most abundant Phyla belong to Pseudomonadota, Thermotogota, Deinococcota, Calditrichota, Thermodesulfobacteriota, Spirochaetota, and Armatimonadota. The classification of the representative thermophilic bacterial genera that were identified (Fig. 1b) is in good agreement with similar studies on hot springs soil samples (Sharp et al. 2014; Lavrentyeva et al. 2018; Kochetkova et al. 2020; Shu and Huang 2022; Peach et al. 2022).

Prior to testing the functional properties of Ami1, a bioinformatic and in silico investigation was conducted with the initial findings supporting the prophagic origin of the encoding gene. Prophages represent a valuable source of new genes, which are often associated with unique catalytic and functional features. Therefore, from a biotechnology point of view, prophages have attracted significant attention as they can expand the repertoire of available resources for the identification and discovery of novel endolysin sequences (Premetis et al. 2023b). Analysis of the primary sequence of Ami1 uncovered interesting features. The enzyme is a clear example of modular architecture by assembling a catalytic and a CWBD. Gram-positive bacteria endolysins have a modular structure with cell wall-binding domains and enzymatically active domains. On the other hand, gram-negative bacteria use an external membrane barrier, making endolysins from host-specific bacteriophages small, globular proteins without CWBDs (Dams and Briers 2019).

Ami1 consists of two distinct structural domains, an NALAA-2 domain and a LysM. The amidase domain allows breaking of the amide bond that links the N-acetyl muramic acid of glycan strands to the L-alanine residue of the peptide stems, common in most bacterial cell wall peptidoglycans. The LysM region is one of the most common cell wall binding domains that can be found in endolysins and is located either at the N- or the C-terminus (Buist et al. 2008). The NALAA-2 enzymes are Zn^2+^-dependent (E.C. 3.5.1.28) and catalyze the hydrolysis of the amide bond between *N*-acetylmuramic acid (NAM) and an L-amino acid (preferably L-Ala) in the PG structure. The Zn^2+^ binding site is formed by the presence of two conserved His and one Cys or Asp residues (Martínez-Caballero et al. 2013; Alcorlo et al. 2017; Broendum et al. 2018). A common feature of NALAA-2 enzymes is their structural flexibility, as they undergo induced-fit conformational changes, adopting an open/close conformation upon substrate binding (Liepinsh et al. 2003; Carrasco-López et al. 2011). The LysM domain is a widely occurring structural unit in enzymes that bind PG in bacteria or chitin in eukaryotes. It recognizes *N*-acetylglucosamine and is typically composed of 44–65 amino acids. In general, enzymes involved in the hydrolysis of bacterial PG frequently contain CWBDs that contribute to cell wall binding, thus facilitating catalysis (Steen et al. 2005; Shao et al. 2012; Bosma et al. 2006).

Ami1 was expressed in *E. coli* and purified to homogeneity by metal-chelate affinity chromatography. Biochemical analysis revealed that Ami1 displayed significant lytic activity against a range of gram-positive and negative bacterial species. Endolysins can combat both gram-positive and gram-negative bacteria because of their unique structures and modes of action. However, because gram-negative bacteria have an outer membrane that prevents exogenously applied enzymes from penetrating, the use of endolysins against these bacteria is restricted. Endolysins that display intrinsic outer membrane permeability have been reported and show broad range of activity against targeted gram-negative bacteria (Guo et al. 2017; Wu et al. 2018; Plotka et al. 2019). Such endolysins have an amphipathic alpha-helical structure at the C-terminus that resembles the antimicrobial peptide (AMP) and facilitates the passage of the entire protein through the outer membrane (Gutiérrez and Briers 2021). The observed broad specificity of Ami1 is probably the consequence of the LysM domain since the majority of enzymes that contain this region display a broad spectrum of specificity (Buist et al. 2008).

The enzyme is effective towards both inactivated as well as live bacterial cells. Interestingly, among all bacterial species examined, Ami1 showed a preference towards two key bacterial pathogens, *S. aureus* and *S. epidermidis*, that have become the most important cause of nosocomial infections in recent years (Chessa et al. 2016).

Endolysins are eco-friendly antimicrobials with rapid action and low bacterial resistance risk. However, their low stability can hinder their clinical use. Endolysins from mesophilic bacteria infecting phages generally lose their hydrolytic activity above 45–55 °C (Premetis et al. 2023a; Abouhmad et al. 2020; Urdániz et al. 2022). Researchers have attempted to fuse thermophile lytic enzymes with CWBDs to create thermostable endolysins (Swift et al. 2019). Alternative methods include structure-based point mutations, in silico design of chimeric endolysins, and stabilizer addition to improve thermostability (Heselpoth et al. 2015; Love et al. 2021). The melting temperature (*T*_*m*_) of Ami1 indicates high thermal stability, compared with other known bacteriophage endolysins (Linden et al. 2015; Bustamante et al. 2012; Abouhmad et al. 2020; Urdániz et al. 2022), suggesting that Ami1 can be classified into thermostable enzymes.

Overall, Ami1 by possessing broad lytic activities and high stability can be considered as a promising antibacterial agent. Ami1 could be further exploited as a candidate molecule for the treatment of infections caused by *S. aureus* and *S. epidermidis*. Further experimental studies are essential for a more detailed characterization of this new amidase.

## Supplementary information

Below is the link to the electronic supplementary material.

Supplementary file1 (PDF 381 KB)

## Data Availability

Sequencing data were deposited in National Center for Biotechnology Information (NCBI) and can be accessed via BioProject ID PRJNA997949. The Ami1 gene sequence was deposited at the GenBank under the accession number OQ725913. All other data supporting the findings of this study are available within the paper. Plasmids encoding the enzyme constructs studied in this work are available upon reasonable request.
