# Supplementary material for: Metagenomic analysis of hot spring soil for mining a novel thermostable enzybiotic
Source: Appl Microbiol Biotechnol. 2024 Jan 22;108:163. doi: 10.1007/s00253-023-12979-2 (PMC10803476; doi:10.1007/s00253-023-12979-2)
Supplement: Supplementary file 1 [file 253_2023_12979_MOESM1_ESM.pdf]

Applied Microbiology and Biotechnology

Metagenomics analysis of hot spring soil for mining a novel thermostable enzybiotic

Panagiota D. Pantiora[#], Nikolaos D. Georgakis[#], Georgios E. Premetis and Nikolaos E. Labrou[1]*

Laboratory of Enzyme Technology, Department of Biotechnology, School of Applied Biology and Biotechnology, Agricultural University of Athens, 75 Iera Odos Street, GR-11855-Athens, Greece

[#]Equal contribution

*To whom correspondence should be addressed: Nikolaos E. Labrou,

E-mail: Lambrou@aua.gr

Tel & Fax: +305294308

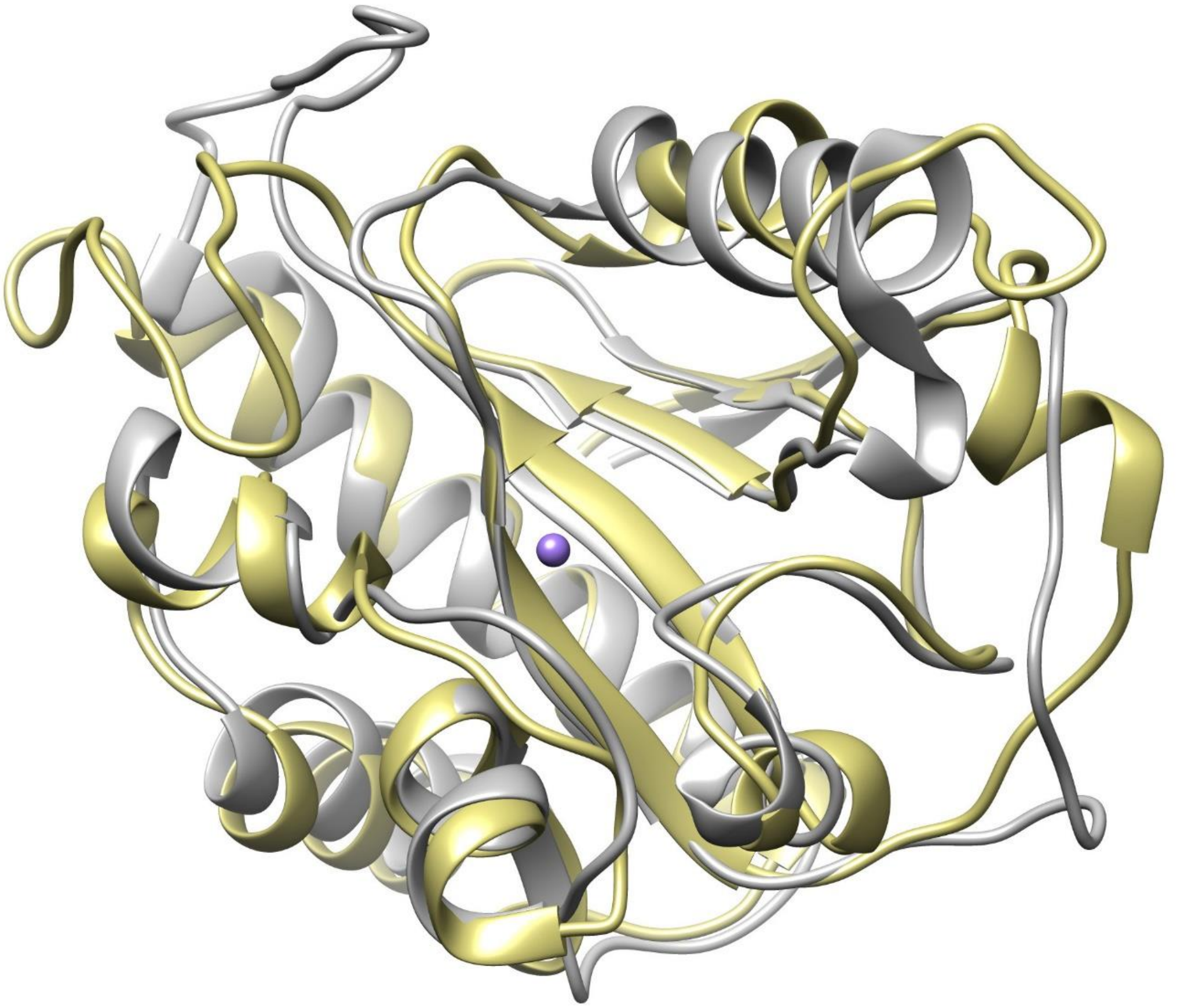

Figure S1. Superposition of the NALAA-2 domain structures, predicted by iTASSER (grey color) (Yang and Zhang, 2015) and AlphaFold (green color) (Jumper et al., 2021). The calculated RMSD is 1.857 angstrom. The figure was created using the program UCSF Chimera 1.16.
